# Supplementary material for: Glycemic fluctuations, fatigue, and sleep disturbances in type 2 diabetes during ramadan fasting: A cross-sectional study
Source: PLoS One. 2025 Mar 5;20(3):e0312356. doi: 10.1371/journal.pone.0312356 (PMC11882071; doi:10.1371/journal.pone.0312356)
Supplement: S2 Table — (DOCX) [file pone.0312356.s002.docx]

S2 Table. Additional questions related experience fatigue and sleep quality among type 2 diabetes during Ramadan fasting in health care settings

| **Questions** |
| --- |
| Do you feel fatigue during Ramadan? |
| How long have you felt fatigue during Ramadan? |
| Are you experiencing sleep disturbances during Ramadan? |
| How long have you had sleep disturbance during Ramadan? |
| Have you ever discussed “the feeling of tired” or “fatigue” with your physician during Ramadan? |
| Never |
| Rarely |
| Sometimes |
| Often |
| Always |
| Have you ever discussed “the experiencing of sleep disturbance” with your physician during Ramadan? |
| Never |
| Rarely |
| Sometimes |
| Often |
| Always |
| Whether your fatigue level has been measured by your physician during Ramadan? |
| Never |
| Rarely |
| Sometimes |
| Often |
| Always |
| Has your sleep quality level been measured by your physician during Ramadan? |
| Never |
| Rarely |
| Sometimes |
| Often |
| Always |
| Has your fatigue been treated by physician during Ramadan? |
| Never |
| Rarely |
| Sometimes |
| Often |
| Always |
| Has your sleep disturbance been treated by a physician during Ramadan? |
| Never |
| Rarely |
| Sometimes |
| Often |
| Always |
