# Supplementary material for: Glycemic fluctuations, fatigue, and sleep disturbances in type 2 diabetes during ramadan fasting: A cross-sectional study
Source: PLoS One. 2025 Mar 5;20(3):e0312356. doi: 10.1371/journal.pone.0312356 (PMC11882071; doi:10.1371/journal.pone.0312356)
Supplement: S3 Table — (DOCX) [file pone.0312356.s003.docx]

S3 Table. M**eal timing and frequency** during Ramadan fasting

| Meal group | Definition |
| --- | --- |
| Two meals | Participants consume **two main meals** during Ramadan fasting: one pre-dawn (Suhoor) and one post-sunset (Iftar). There are no additional snacks or meals consumed between Iftar and Suhoor. |
| Three Meals | Participants consume **three meals** during Ramadan fasting: one pre-dawn (Suhoor), one post-sunset (Iftar), and a **small snack** or meal after Iftar (post-Iftar snack). This pattern helps spread nutrient intake throughout the evening. |
| Irregular meal pattern | Participants have **inconsistent meal timing or frequency** during Ramadan, such as skipping Suhoor or Iftar, or eating at unpredictable times. This group may also have irregular meal portions or frequency, leading to an unstructured eating pattern. |
