# Supplementary material for: Glycemic fluctuations, fatigue, and sleep disturbances in type 2 diabetes during ramadan fasting: A cross-sectional study
Source: PLoS One. 2025 Mar 5;20(3):e0312356. doi: 10.1371/journal.pone.0312356 (PMC11882071; doi:10.1371/journal.pone.0312356)
Supplement: S4 Table — (DOCX) [file pone.0312356.s004.docx]

S4 Table. Dietary patterns during Ramadan fasting

| Dietary patterns group | Definition |
| --- | --- |
| Low glycemic index foods | Participants who consume **low glycemic index foods** (such as whole grains, legumes, non-starchy vegetables, and lean proteins) during both Iftar (meal after sunset), Suhoor (pre-dawn meal), and/or post-Iftar snacks. |
| Random eating habits | Participants with **random eating habits**, characterized by irregular meal timing, consumption of high-GI foods (e.g., sweets, fried foods), and imbalanced portions of macronutrients (carbs, fats, proteins) during both Iftar, Suhoor, and/or post-Iftar snacks. |
